# Application of comprehensive unit-based safety program model to improve chemotherapy-induced nausea and vomiting in patients with ovarian cancer: a retrospective study

**DOI:** 10.1186/s13048-023-01230-1

**Published:** 2023-07-20

**Authors:** Aihua Liu, Min Li, Zhuo Liu, Xinxin Xie, Wen Yao, Jingmin Wang, Tingting Zhao, Jie Yang

**Affiliations:** 1 Department of Gynaecology, The First Affiliated Hospital of Anhui Medical University, No.120 Wan Shui Road, Shu Shan District, Hefei, Anhui Province 230022 China; 2 Department of nursing management, The First Affiliated Hospital of Anhui Medical University, Hefei, Anhui Province China

**Keywords:** Comprehensive unit-based safety program, Chemotherapy-induced nausea and vomiting, Ovarian cancer, Quality of life, Anxiety, Depression

## Abstract

**Background:**

To explore the effect of intervention programs constructed under the guidance of the comprehensive unit-based safety program (CUSP) model on chemotherapy-induced nausea and vomiting (CINV) in patients with ovarian cancer.

**Method:**

According to the time of admission, 90 ovarian cancer chemotherapy patients in the first affiliated Hospital of Anhui Medical University from June 2019 to September 2020 were divided into an intervention group and a control group with 45 cases each. Both groups of patients received routine intervention, and the intervention group implemented the CUSP program on this basis. The intervention lasted 8 months. Before and after the intervention, the patients in the ward were used the Multinational Association of Supportive Care in Cancer (MASCC) Antiemesis Tool, the Functional Living Index-Emesis (FLIE), and the Hospital Anxiety and Depression Scale (HADS) for the effect evaluation.

**Results:**

After the intervention, the degree of nausea and vomiting frequency in the intervention group were significantly lower than that in the control group, especially the degree of nausea in the delayed phase (*P* < 0.05). The score of the functional living index-emesis in the intervention group was significantly higher than that in the control group (*P* < 0.05), and the anxiety and depression in the intervention group were significantly relieved compared to the control group (*P* < 0.05).

**Conclusion:**

The intervention program guided by the CUSP model can significantly alleviate patients’ nausea and vomiting, improve the quality of life, and relieve anxiety and depression. The CUSP model is suitable for clinical practice and has guiding significance for clinical work.

## Background

Ovarian cancer was the most malignant gynecological tumor. In tumors of the female reproductive system in China, the incidence rate of ovarian cancer was the third, and the mortality rate was the first [1]. Due to the lack of effective screening methods and difficulties in early diagnosis, 70% of ovarian cancer patients were already in advanced stage at the time of initial diagnosis, 50–70% relapse within 2 years after treatment, and the 5-year survival rate was less than 30%[2]. Ovary tumor rebulking operation combined with adjuvant chemotherapy was the main treatment [3]. For advanced patients, chemotherapy is an important means to improve the prognosis and clinical outcome. However, chemotherapy has many side effects, such as chemotherapy-induced nausea and vomiting (CINV). CINV refers to the nausea and vomiting caused by the use of chemotherapy drugs, which is the most common adverse reaction in the chemotherapy of tumor patients [4]. Related studies showed that 58.1% of ovarian cancer patients experienced nausea and 31.0% vomited after chemotherapy [5]. Although the occurrence of CINV has been improved with the application of antiemetic drugs, nausea and vomiting in about 40% of patients still cannot be effectively alleviated [6]. CINV affects the safety of the treatment process, reduces the quality of life of patients, and forces the reduction of chemotherapy dose or even termination of treatment [7]. It is necessary to explore the intervention to improve CINV in ovarian cancer patients.

At present, CINV interventions can be divided into two categories, one is drug intervention and the other is non-drug intervention, including sports training, diet intervention, music therapy, traditional Chinese medicine treatment and so on [8]. However, the intervention methods are not systematic and comprehensive, lack of multidisciplinary cooperation, and the results are uneven. There were also few interventions for ovarian cancer patients. Comprehensive Unit-based Safety Program (CUSP) which was funded predominantly by the Agency for Healthcare Research and Quality (AHRQ), was a theoretical model to ensure patient safety and reduce the incidence of adverse events. Through the formation of multidisciplinary teams and centralized CUSP meetings, patient safety practices are systematically carried out to reduce or eliminate the possibility of patients being harmed in hospitals [9]. CUSP had been successfully applied to reduce surgical site infection abroad, but few reports had been reported in China. This study intends to intervene CINV in ovarian cancer patients under the guidance of CUSP model, in order to improve the status quo of CINV in patients and provide experience for clinical workers to intervene CINV in ovarian cancer patients.

## Methods

### Study participants

Patients who needed chemotherapy for ovarian cancer from June 2019 to September 2020 and were willing to participate in the program were selected in the Department of Gynaecology, the first affiliated Hospital of Anhui Medical University. All patients were required to sign informed consent prior to participating in the program. This study was approved by the Hospital Ethics Committee of the first affiliated Hospital of Anhui Medical University, Ethics No. : PJ2021-06-21.

The inclusion criteria of the patients were as follows: ①patients with pathologically confirmed ovarian cancer; ②Karnofsky performance status (KPS) ≥ 70; ③age > 18-years-old; ④patients with moderate/high risk of emetic medication, in which the risk level of chemotherapy drug-induced vomiting developed by the Multinational Association of Supportive Care in Cancer (MASCC) [10]; ⑤the third chemotherapy of this course is planned; ⑥informed consent and voluntary participation. The exclusion criteria of the patients were: ①patients with chemotherapy and radiotherapy; ②patients with intestinal obstruction and other gastrointestinal diseases or a history of such diseases; ③patients with severe liver and kidney dysfunction; ④unable to accept intervention or transferred to another hospital for treatment due to disease factors.

The formula n = 2(u_α_+ u_β_)^2^P(1-P)/(P_1_-P_0_)^2^ was used to calculate the sample size. P_1_ and P_0_ represent effective rates with and without intervention, respectively. Accordding to the literature search [11], P1 = 85%, P0 = 54%. P=( P_1_ + P_0_)/2. The significant level α was 0.05, u_α_=1.96. Type II error rate β was 0.1, u_β_=1.282. Considering the 10% loss to follow-up rate, a total of 90 patients were selected as the sample size. 45 patients from June 2019 to January 2020 were selected as the control group, and 45 patients from February 2020 to September 2020 were selected as the intervention group.

### Study design and intervention method

#### Routine chemotherapy nursing

The control group received routine chemotherapy nursing. Before chemotherapy, antiemetic drugs were given according to the doctor’s advice, and the purpose and precautions of drug use were informed. Meanwhile, CINV knowledge education was conducted. In the course of chemotherapy, the adverse reactions of chemotherapy should be closely observed, and the severity of nausea and vomiting should be evaluated. When the nausea and vomiting of the patient affected eating, the antiemetic program should be changed if necessary by timely communication with the doctor. Informing the patient of matters needing attention at home after chemotherapy. Patients will be followed up by nurses post-discharge, so that nurses can timely grasp the situation of nausea and vomiting. Patients were advised to see a doctor in time for any discomfort post-discharge.

#### Chemotherapy nursing based on CUSP model

On the basis of the control group, the intervention group carried out multidisciplinary collaborative management based on the guidance of CUSP model, with the establishment of safety culture in the ward as the core and the improvement of CINV status of patients as the purpose.

##### **Study CUSP**

Through the web site https://www.ahrq.gov/hai/cusp to download information about CUSP. Learn the CUSP toolkit. Foreign language professionals and medical professionals with overseas study experience were invited to translate and organize the materials. The materials were used to train medical staff in wards.

##### **Build a multidisciplinary CUSP team**

The team includes 1 person in charge of nursing management department, 1 director of the ward, 1 head nurse (master’s degree), 1 doctors (doctor’s degree), and 5 nurses (1 master’s degree, 4 bachelor’s degree). Nursing management staff was responsible for guiding the implementation of the program and providing resource support. Director of the ward coordinated the cooperation of medical staff. Ward doctors were responsible for the treatment of medical events and related medication knowledge training. The head nurse of the ward had received professional training of CUSP program. As the CUSP project leader, she supervised the implementation of the program, project design, quality control, organized and participated in CUSP team meetings and CUSP knowledge training. The ward nurses were responsible for relevant nursing content training and the implementation of the plan, investigating and tracking the patient’s situation, collecting and sorting of data.

##### Look for defects

①The head nurse of the ward organized CUSP meetings and invited administrative staff to participate. CUSP team members brainstormed about events in the ward that adversely affected patient safety. Under the guidance of the CUSP toolkit, each team participant was required to answer the following questions: (a) What do you think were the current security problems in the ward? (b) Which problem do you think had the most adverse effect on patients? (c) What measures do you think could be taken to improve it? (d) What indicators do you think could be used to evaluate the effect of intervention? ②Baseline investigation was conducted on the occurrence of adverse events in the ward. Through investigation to understand the current situation of safety culture in the ward, the existing deficiencies, and the status quo and influencing factors of adverse events. ③Through literature review to identify the existing problems and severity. Through the above steps, the main defects were finally identified as follows: ①Severe nausea and vomiting occurred during and after chemotherapy in patients with ovarian cancer. ②Severe nausea and vomiting resulted in psychological problems, which affected treatment compliance and prognosis.

###### Multidisciplinary collaborative intervention

Stage 1: Team training. One project per week, one hour each time. The training content was as follows: ①CINV medication safety knowledge training. The training was carried out by on-site teaching and wechat group pushing relevant knowledge [12]. Team members reported the action principle, common side effects, dosage, interval, course of treatment, follow-up indicators of commonly used chemotherapy drugs in ovarian cancer patients and the research status of this drug in recent 5 years by Microsoft Office PowerPoint (PPT). ②CINV diet knowledge training. First, ward head nurse explains the importance of dietary intervention to enhance team members’ awareness. Second, inviting nutrition division expert to undertake specific diet training. The training contents included the basic requirements of balanced diet, dietary characteristics and key points of tumor patients, dietary strategies for nausea and vomiting, etc [13, 14]. ③CINV exercise guidance training. Ward head nurse stated the importance of exercise and explained common exercise patterns for cancer patients. Literature shows that “baduanjin exercise” is helpful for tumor patients to massage the viscera [15]. The training process is combined with the video, and the video QR code is created and sent to the wechat group. ④CINV psychological intervention knowledge training. Nurses with national Psychological consultant certificateIIwere responsible for psychological training. Psychological consultant explained the importance of cancer patients psychological nursing, psychological experience of six stages and nursing measures, common psychological problems and nursing measures, self-regulation of cancer patients such as music therapy and other content [16]. CUSP group members learned the training content together. Questions will be asked in the morning meeting every day, and assessment will be conducted once a month to consolidate the learning effect of team members.

Stage 2: Intervention implementation. ①Developing detailed, easy-to-understand health education manuals with pictures according to the age, education level and cognitive level of patients undergoing chemotherapy. Placing the manuals on the ward publicity board for easy reference by patients and carers. ②Selecting ward meeting room for health education lectures. Lectures will be given by trained CUSP team members and invited experts. The lectures covered the prevention of CINV, such as medication precautions, dietary precautions, exercise precautions and psychological problems, etc. CUSP team members or invited experts answered questions about CINV from patients or carers on site. The “baduanjin exercise” was taught on-site after the meeting. Meetings were held 2–3 times a week, each lasted 40 min to 1 h. ③Before each chemotherapy, the doctors in the ward used the prescription of antiemetic drugs preventively, such as metoclopramide + tropisetron + dexamethasone. For highly emetogenic chemotherapy drugs, the combination of aprepitant + tropisetron + dexamethasone would be used. ④On the morning of chemotherapy, patients were advised to eat light and digestible food and avoid fasting. It was advisable to eat 1/2 of the daily amount for breakfast and 2/3 of the daily amount for lunch and dinner. Appropriately increase foods with high calorie, high nutrition and high vitamins, such as eggs, fish and shrimp, fruits and vegetables, animal offal, etc., and avoid eating foods that are cold, irritating, hot and easy to produce gas at the same time, such as onions, leeks, raw garlic and radishes. If patients had vomiting symptoms, they still need to insist on eating, which could neutralize gastric acid, promote intestinal peristalsis and reduce vomiting. Patients were also encouraged to drink more water to ensure that the daily urine output was about 2000 mL. ⑤Every morning before the morning meeting, CUSP team members leaded chemotherapy patients to do “baduanjin exercise” for 10 min each time, and recorded “baduanjin exercise” videos sent to patients and carers. ⑥When patients were discharged from the hospital, the doctors prescribed antiemetic drugs and told the patient to take metoclopramide tablets 5 mg orally, three times a day. ⑦Pay attention to extended care. CUSP team members established CINV communication wechat group, and regularly sent the electronic version of CINV related knowledge and “baduanjin exercise” videos to the group and encouraged patients to exercise regularly every morning. At the same time, team members paid attention to the occurrence of CINV expressed by patients in the group and and urged patients to adhere to taking antiemetic drugs every day. Telephone follow-up was conducted once a week to understand the occurrence of delayed CINV during the patient’s stay at home and gave timely guidance.

Stage 3: Assessment. After the intervention, the patients’ nausea and vomiting were evaluated to judge the effect of the implementation of CUSP. The head nurse of the ward convened a CUSP meeting to discuss the existing shortcomings and the advantages of the intervention again to improve the work ahead. To ensure that the whole CUSP program was implemented in a cycle of constantly finding defects and improving interventions with patient as the center.

### Effectiveness evaluation tool

#### Patient general demographic data questionnaire

Through literature review and CUSP conference discussion, our team prepared the patient general demographic data questionnaire. The questionnaire included the patient’s age, education level, place of residence, type of tumor and chemotherapy regimen and so on.

#### MASCC Antiemesis Tool

The tool, developed by the Multi-country Collaboration on Cancer Support therapy [17], was a self-rating scale with 8 items in 2 dimensions. Items 1, 3, 5, and 7 evaluated whether nausea and vomiting occurred. Items 2 and 6 recorded the frequency of vomiting. Items 4 and 8 assessed the severity of nausea. Likert 10-level scoring method was used, ranging from no nausea to extreme nausea. Cronbach’s α coefficient was 0.71.

#### Functional living index-Emesis (FLIE)

The scale was developed by Lindley et al. to evaluate the impact of chemotherapy-related nausea and vomiting on patients’ quality of life, including two dimensions of nausea and vomiting, with 9 items for each [18]. Likert 7-level scoring method was adopted, with 1 point representing serious impact and 7 points representing no impact. Each item was scored accumulative. A total score of ≥ 108 indicates no effect on patients, while a score of < 108 indicates an effect on patients. The scale internal reliability and structure validity of the scale were 0.79 and 0.74 ~ 0.97, respectively.

#### Hospital anxiety and Depression Scale (HADS)

The scale was compiled by Zigmond and Snaith in 1983 and mainly applied to the investigation of anxiety and depression among patients in general hospitals. It contained 2 dimensions and 14 items. 7 items rated anxiety (HADS-A) and 7 rated depression (HADS-B). Each item was graded on a scale of 0 to 3. It had been reported that a score of 9 was the critical point of each dimension. A score of > 9 was considered as positive for the existence of anxiety and depression symptoms, while a score of < 9 was considered as negative [19]. Cronbach’s α of subscale and total scale were 0.879, 0.806 and 0.806.

### Quality Control

This study was called on by the head nurse who had been trained by CUSP project. The whole process was monitored and the team members were divided, so that the research process was carried out in strict accordance with the steps of CUSP project implementation. The intervention time was 8 months, and all the intervention contents were guaranteed to be completed within 8 months. In order to reduce the influence of anticipatory nausea and vomiting on the study, the chemotherapy course of the patients was controlled at the time of enrollment, and the patients were required to receive the third chemotherapy of this course. In order to ensure the quality of the questionnaire, 5 nurses were assigned to collect the questionnaire data, and centralized training was conducted before the collection to unify the standards. 4 of them with bachelor’s degree went into the ward to issue questionnaires and told the patients the matters needing attention. If the patients had difficulty in filling out questionnaires, the nurses would fill them out by asking. Data on delayed nausea and vomiting were collected through patients’ or carers’ records and telephone follow-up. Another nurse with master’s degrees was responsible for checking the logic of the questionnaire and other quality issues. In order to reduce the influence of chemotherapy course on CINV degree of patients, the questionnaire filling time was strictly set. The filling time before intervention was before the third chemotherapy of this course, and the filling time after intervention was after the sixth chemotherapy. The collected data was checked and entered into the system by 2 nurses.

### Statistical analysis

IBM SPSS 23.0 was used to analyze the data. The measurement data conforming to normal distribution were described by mean ± standard deviation. The independent sample t-test was used for the comparison between the two groups of means. The measurement data that did not conform to normal distribution were represented by median, and the comparison between the two groups was performed by nonparametric Mann-Whitney U test. The enumeration data were expressed as a percentage, and the chi-square test was used for the comparison of the two groups. *P* < 0.05 was considered a significant difference.

## Results

### Comparison of general demographic data of patients

There was no significant difference in general demographic data between the intervention group and the control group in baseline investigation (*P* > 0.05), indicating comparability, as shown in Table 1.

**Table 1:** Comparison of general demographic data between the two groups

Variables	Categories	The intervention group (n = 45)	The control group (n = 45)	*T* /${ \chi }^{2}$	*P*
Age		54.87 ± 10.51	55.93 ± 12.92	-0.430	0.668
Level of education	Primary or below	31	33	2.196	0.533
	Junior high	9	9		
	Senior high / technical secondary	4	1		
	Undergraduate / Junior college or above	1	2		
Place of residence	Rural	33	36	0.559	0.455
	Urban	12	9		
Employment	Employed	12	8	1.029	0.310
	Unemployed	33	37		
Vomiting of pregnancy	Yes	34	33	0.058	0.809
	No	11	12		
Pathological type	Epithelial carcinoma of the ovary	43	40	1.442	0.486
	Malignant germ cell tumor of ovary	1	3		
	Malignant ovarian cord-stromal tumor	1	2		
Relapse	No	31	34	0.498	0.480
	Yes	14	11		
Chemotherapy regimens	TP regimens^a^	24	23	1.807	0.613
	EP regimens^b^	2	5		
	DC regimens^c^	5	3		
	Others^d^	14	14		
The score of KPS^e^	70–90	10	13	0.526	0.468
	>90	35	32		

^a^: Cis-platinum + Paclitaxel; ^b^: Etoposide + Paclitaxel; ^c^: Docetaxel + Carboplatin;

^d^: Doxorubicin hydrochloride + Carboplatin; Topotecan + Bevacizumab; Paclitaxel + Carboplatin _+_ Bevacizumab; Apatinib mesylate + Etoposide;

^e^: Karnofsky performance status

### Comparison of nausea degree and vomiting frequency in acute and delayed periods between the two groups before and after intervention

After intervention, the nausea and vomiting of patients in the intervention group were significantly relieved compared with those in the control group, and the difference was statistically significant (*P* < 0.05), as shown in Tables 2 and 3.

**Table 2:** Comparison of vomiting frequency and nausea degree in acute phase between two groups

Variables	Intentional analysis				Per-protocol analysis*			
	The intervention group(n = 45)	The control group(n = 45)	*Z*	*P*	The intervention group(n = 41)	The control group(n = 41)	*Z*	*P*
Vomiting frequency (before intervention)	1.0(1.0,2.0)	1.0(1.0,2.0)	-0.540	0.589	1.0(1.0,2.0)	1.0(1.0,2.0)	-0.231	0.817
Vomiting frequency (after intervention)	1.0(0.0,1.0)	1.0(0.5,1.0)	-2.770	0.006	1.0(0.0,1.0)	1.0(0.0,1.0)	-2.075	0.038
Nausea degree (before intervention)	2.0(1.0,3.0)	2.0(1.0,2.5)	-1.587	0.113	2.0(1.0,2.5)	1.0(1.0,2.5)	-1.525	0.127
Nausea degree (after intervention)	1.0(0.0,1.0)	1.0(1.0,2.0)	-3.400	0.001	1.0(0.0,1.5)	1.0(1.0,2.0)	-2.375	0.018

*: Per-protocol analysis was performed on patients who completed the entire intervention. In the intervention group, 4 cases were excluded, including 2 cases transferred to hospital and 2 cases transferred to department. In the control group, 1 case was transferred to another hospital and 3 cases to another department

**Table 3:** Comparison of vomiting frequency and nausea degree in delayed phase between two groups

Variables	Intentional analysis				Per-protocol analysis			
	The intervention group(n = 45)	The control group(n = 45)	*Z*	*P*	The intervention group(n = 41)	The control group(n = 41)	*Z*	*P*
Vomiting frequency (before intervention)	5.0(4.0,5.0)	5.0(2.0,5.5)	-0.391	0.695	5.0(4.0,5.0)	5.0(2.0,5.5)	-0.798	0.425
Vomiting frequency (after intervention)	3.0(2.0,3.0)	4.0(2.0,5.0)	-3.259	0.001	3.0(1.5,3.5)	4.0(2.0,5.0)	-2.712	0.007
Nausea degree (before intervention)	4.0(4.0,5.0)	5.0(2.0,6.0)	-0.723	0.470	4.0(3.5,5.0)	5.0(2.0,6.0)	-0.559	0.576
Nausea degree (after intervention)	3.0(2.0,4.0)	5.0(2.0,5.5)	-1.989	0.047	3.0(2.0,4.0)	4.0(1.5,5.0)	-2.313	0.021

### Comparison of functional living index scores between two groups before and after intervention

After intervention, the functional living index scores of patients in the intervention group were significantly higher than those in the control group, and the difference was statistically significant (*P* < 0.05), as shown in Table 4.

**Table 4:** Comparison of functional living index scores between two groups before and after intervention

Variables	Intentional analysis				Per-protocol analysis			
	The intervention group(n = 45)	The control group(n = 45)	*t*	*P*	The intervention group(n = 41)	The control group(n = 41)	*t*	*P*
Nausea dimensions (before intervention)	34.69 ± 7.95	36.33 ± 9.20	-0.907	0.367	35.59 ± 7.74	36.83 ± 9.40	-0.654	0.515
Nausea dimensions (after intervention)	44.56 ± 7.83	36.93 ± 9.31	4.206	0.000	44.56 ± 8.21	37.49 ± 9.49	3.609	0.001
Vomiting dimensions (before intervention)	34.29 ± 7.82	35.38 ± 9.64	-0.588	0.558	35.22 ± 7.50	35.85 ± 9.89	-0.327	0.744
Vomiting dimensions (after intervention)	43.07 ± 7.03	35.82 ± 9.41	4.140	0.000	43.07 ± 7.37	36.24 ± 9.66	3.597	0.001
Total dimensions (before intervention)	68.98 ± 15.42	71.71 ± 18.68	-0.757	0.451	70.80 ± 14.84	72.68 ± 19.13	-0.497	0.621
Total dimensions (after intervention)	87.10 ± 14.86	72.76 ± 18.10	4.124	0.000	87.05 ± 15.58	73.73 ± 18.37	3.540	0.001

### Comparison of anxiety and depression scores between two groups before and after intervention

After intervention, anxiety scores, depression scores and total scores of patients in the intervention group were improved compared with those in the control group, and the differences were statistically significant (*P* < 0.05), as shown in Table 5.

**Table 5:** Comparison of anxiety and depression scores between two groups before and after intervention

Variables	Intentional analysis				Per-protocol analysis			
	The intervention group(n = 45)	The control group(n = 45)	*t*	*P*	The intervention group(n = 41)	The control group(n = 41)	*t*	*P*
anxiety dimensions (before intervention)	12.69 ± 2.74	13.56 ± 3.10	-0.907	0.367	12.59 ± 2.67	13.41 ± 3.17	-0.654	0.515
anxiety dimensions (after intervention)	7.02 ± 1.84	13.38 ± 3.36	4.206	0.000	7.02 ± 1.93	13.29 ± 3.44	3.609	0.001
depression dimensions (before intervention)	12.53 ± 2.26	12.62 ± 2.81	-0.588	0.558	12.41 ± 2.29	12.61 ± 2.90	-0.327	0.744
depression dimensions (after intervention)	7.07 ± 1.97	12.12 ± 3.54	4.140	0.000	7.07 ± 2.07	12.12 ± 3.72	3.597	0.001
Total dimensions (before intervention)	25.22 ± 4.76	26.18 ± 5.54	-0.757	0.451	25.00 ± 4.73	26.02 ± 5.72	-0.497	0.621
Total dimensions (after intervention)	14.10 ± 3.34	25.50 ± 6.17	4.124	0.000	14.10 ± 3.51	25.41 ± 6.43	3.540	0.001

## Discussion

### CUSP program can improve nausea and vomiting in acute and delayed phases in ovarian cancer patients

This study showed that before intervention, there was no difference in nausea and vomiting in the acute and delayed stages between the two groups, but after intervention, the nausea degree and vomiting frequency of the intervention group were significantly lower than that of the control group, indicating that the implementation of CUSP intervention program could effectively improve the symptoms of nausea and vomiting in patients. Studies had shown that CUSP could effectively reduce the infection rate at the surgical site [20], the incidence of Ventilator associated pneumonia [21], catheter-related infection [22]. CUSP could also be used to prevent patients from falling down [23], reduce the occurrence of adverse events during patient transfer [24] and reduce nosocomial infection [25]. Therefore, CUSP could significantly improve adverse events in hospitals and positively affect patients’ safety and quality of life. In addition, this study found that the acute and delayed nausea and vomiting of patients in the intervention group were improved after intervention. However, acute nausea and vomiting also decreased in the control group compared with before intervention. The reason might be that the blood concentration of chemotherapy drugs was too high in patients with acute nausea and vomiting before intervention, resulting in significant emetic effect. After several times of chemotherapy, patients’ tolerance to nausea and vomiting was strengthened, which also led to the decrease of nausea and vomiting. The results of this study showed that the degree of delayed nausea and vomiting in the control group was not significantly decreased before and after intervention, indicating that CUSP intervention program had a significant effect on delayed nausea and vomiting in chemotherapy patients. This study innovatively applied CUSP to patients undergoing chemotherapy, and proved that the implementation of CUSP program had significant effect on improving CINV of patients undergoing chemotherapy for ovarian cancer. The program emphasized teamwork, constantly finding defects and improving patients’ problems, and achieving dynamic supervision and intervention, which could not only provide reference for clinical workers to construct intervention programs, but also expanded the research field of CUSP. Studies had shown that the effect of CUSP in preventing adverse events could last several years after the implementation of the program [26], which indicated that the continuous effect of benefits after the implementation of CUSP program was very strong.

### CUSP program can improve quality of life in patients with ovarian cancer

Chemotherapy could improve the survival rate of patients, but it could not ensure the quality of life of patients, especially the side effects of chemotherapy such as nausea and vomiting seriously affect the quality of life of patients. The results of this study showed that the patients’ functional living index-emesis score was not high, indicating that CINV had a great impact on patients’ life, which was similar to the results of relevant studies [27, 28]. After the implementation of CUSP program, the score of patients’ functional living index-emesis was significantly improved in the intervention group, which might be related to the comprehensive coverage of patients’ needs in this program and the implementation of evidence-based interventions including diet, drugs, exercise and psychology, which could significantly improve CINV of patients [8, 29]. In this study, although the scores of nausea and vomiting functional living index of patients were significantly improved compared with before the intervention, the scores were still not high, indicating that CINV was the influence of long-term chemotherapy for patients, and it was difficult to achieve effective relief only by a certain period of intervention, requiring long-term attention and management. However, CUSP was a circular process, which could continuously identify risks and search for defects, as well as carried out long-term follow-up management for chemotherapy patients, further demonstrating the value of CUSP in the application of chemotherapy patients.

### CUSP program can effectively improve anxiety and depression in patients with ovarian cancer

Anxiety and depression seriously affect the quality of life of ovarian cancer patients [30]. If patients had anxiety and depression before chemotherapy, the risk of nausea and vomiting after chemotherapy would increase [31]. If the anxiety and depression of patients with CINV were intervened, the emotional state of patients could be significantly improved [32]. Ovarian cancer patients were more prone to anxiety and depression due to their female characteristics, which further aggravated CINV, and CINV in turn aggravated anxiety and depression [33]. Therefore, patients’ anxiety and depression should be one of the key factors for health educators to consider. In this study, the anxiety and depression in the intervention group were significantly relieved after the intervention, indicating that the intervention program was effective for patients’ anxiety and depression. The reason might be that during the CUSP team meeting, the team members put forward important clinical experience on the patient’s anxiety and depression, and at the same time, the team members summarized the current literature and proposed the psychological intervention of CUSP program, such as music therapy, etc. Combined with clinical experience and evidence to ensure the effectiveness of the intervention program. In addition, CUSP required team members to communicate more with patients to understand their treatment status, which increased the opportunities for doctors and nurses to contact patients, timely answer questions, and relieve patients’ psychological pressure.

## Conclusions

This study developed an intervention program for CINV in ovarian cancer patients under the guidance of CUSP model. The results of this study showed that CUSP could improve the status of nausea and vomiting in ovarian cancer patients, improve the quality of life, and relieve anxiety and depression, indicating that CUSP was an effective, comprehensive and systematic intervention program, which could have beneficial effects on patients in many aspects. However, there were several limitations in this study: ①the sample size was small. The control group and intervention group were recruited at different times, which can easily lead to significant selection bias; ②this study was not strictly a randomized controlled study. Thus, it was suggested to expand the sample size and conduct multicenter randomized controlled trials in the future studies to better evaluate the effectiveness of the intervention program. ③CUSP had its own limitations. CUSP emphasized multidisciplinary teamwork, which required a lot of manpower, material resources and time cost, and its operability and economy were facing challenges. Researchers could further optimize and improve the research design in the later stage to make up for the shortcomings of the project.

## Data Availability

The datasets used and analysed during the current study are available from the corresponding author on reasonable request.

## Abbreviations

CUSP: Comprehensive unit-based safety program
CINV: Chemotherapy-induced nausea and vomiting
AHRQ: Agency for Healthcare Research and Quality
KPS: Karnofsky performance status
MASCC: Multinational Association of Supportive Care in Cancer
PPT: Microsoft Office PowerPoint
FLIE: Functional Living Index-Emesis
HADS: Hospital Anxiety and Depression Scale
TP: Cis-platinum + Paclitaxel
EP: Etoposide + Paclitaxel
DC: Docetaxel + Carboplatin
